# Impact of Vitamin D Status on Cardiometabolic Complications among Children and Adolescents with Type 1 Diabetes Mellitus

**DOI:** 10.4274/jcrpe.2266

**Published:** 2016-03-01

**Authors:** Adnan Al Shaikh, Abdullah M. Al Zahrani

**Affiliations:** 1 King Saud bin Abdul Aziz University for Health Sciences, King Abdulaziz Medical City-Jeddah, Department of Pediatrics, Division of Endocrinology, Jeddah, Kingdom of Saudi Arabia; 2 King Saud bin Abdul Aziz University for Health Sciences, King Abdulaziz Medical City-Jeddah, Department of Family Medicine, Jeddah, Kingdom of Saudi Arabia

**Keywords:** type 1 diabetes mellitus, Vitamin D deficiency, metabolic control Conflict of interest: None declared

## Abstract

**Objective:**

There is an ongoing interest in the relationship between vitamin D status and diabetes control and complications. However, data from Saudi Arabia are limited. To determine the impact of vitamin D status on glycemic control and cardiometabolic complications of children and adolescents with type 1 diabetes mellitus (T1DM) attending a tertiary care diabetes clinic in Saudi Arabia.

**Methods:**

Demographic, clinical, and laboratory data of 301 children and adolescent subjects with T1DM (53.5% females) of a mean age of 13.9 years attending King Abdulaziz Medical City-Jeddah during 2010-2013 were retrospectively collected. Relationships between vitamin D status and frequency of hypoglycemia, hemoglobin A1c (HbA1c) level, body mass index (BMI), blood pressure, and lipid profile were evaluated.

**Results:**

The mean duration of diabetes was 7.7±3.7 years. Mean BMI value was 21.1±4.5 kg/m2 and HbA1c was 9.6±1.9% in both genders. Only 26.2% of the patients had a satisfactory HbA1c level. The mean level of 25-hydroxyvitamin D [25(OH)D] was 35.15 and that of cholesterol was 4.75. Vitamin D deficiency [25(OH)D≤37.5 nmol/L] was detected in 63.6% of the male and 67.7% of the female subjects. In males, it was inversely associated with frequency of hypoglycemia (p<0.01), BMI (p<0.05), diastolic blood pressure (p<0.05), and triglyceride levels (p<0.01), while in females, it was inversely associated with current age (p<0.05), age at diagnosis (p<0.01), and triglyceride levels (p<0.01). No significant correlation between HbA1c and vitamin D deficiency was observed.

**Conclusion:**

Vitamin D deficiency was highly prevalent in our study sample and was found to be associated with frequency of hypoglycemic episodes and with adverse cardiometabolic control.

**WHAT IS ALREADY KNOWN ON THIS TOPIC?**

Vitamin D deficiency is common in Saudi children and has been linked to several cardiometabolic risk factors.

**WHAT THIS STUDY ADDS?**

Vitamin D deficiency is highly prevalent in our study and it is associated with frequent hypoglycemia and adverse cardiometabolic control.

## INTRODUCTION

The current global prevalence of type 1 diabetes mellitus (T1DM) under the age of 15 years is estimated to be around 500 000, with the largest demographics found in Europe and North America (1). Epidemiological data also point to an increased incidence of T1DM globally with a rate of around 3-4% per year and with an age of onset younger than previous estimates (2). These observations were confirmed in developed and developing countries, specifically the US, Latin America, Europe, Australia, India, South-east Asia, and China (2,3,4,5,6,7,8,9). Over the last 3 decades, the incidence rate of T1DM is growing also in Saudi Arabia (10). The most recent reports of incidence of T1DM in Saudi children is 27.5/100 000 (11) and 29/100 000 (12) which are high rates. The prevalence of T1DM in Saudi children and adolescents is 109.5 per 100 000 (13).

Moving on to vitamin D, accumulating evidence in recent years has pointed to a significant link between vitamin D deficiency and auto-immune disease as well as endocrine disorders in children (14). Furthermore, vitamin D deficiency is common in Saudi children and has been linked to several cardiometabolic risk factors outside its conventional role in bone homeostasis (15).

To date, studies on T1DM in the Saudi population are limited. Also, emerging risk factors such as vitamin D deficiency for several chronic diseases have been modestly addressed in this specific population. At present, vitamin D deficiency is highly prevalent among the Saudi T1DM patients, both children and adults (16). A national study by Bin-Abbas et al (17) showed an overall prevalence of vitamin D deficiency in 84% of T1DM children and in 59% of healthy children. In the present study, we aimed to describe the clinical presentation and the level of metabolic control in Saudi children and adolescents with T1DM attending the pediatric endocrine clinic at King Abdulaziz Medical City in Jeddah (KAMC-J) and to determine differences in anthropometric and metabolic indices of those with and without vitamin D deficiency.

## METHODS

The study followed the Helsinki declaration recommendations and was approved by the Institutional Review Board of King Abdullah International Medical Research Center. In this retrospective cross-sectional study, we included all children and adolescents between the ages of 1 and 18 years with known T1DM and regular follow-up for more than 3 months attending the pediatric endocrine clinic at KAMC-J from January 2010 to January 2013. Gender, puberty staging, duration of T1DM, symptoms of early presentation were collected as well as clinical information such as blood pressure (BP) using the Dinamap automated oscillometric device and body mass index (BMI) using Center for Disease Control and Prevention charts. Data on hemoglobin A1c (HbA1c), 25-hydroxyvitamin D [25(OH)D] levels, lipid profile, and thyroid function were also collected from the medical records. We followed the American Diabetes Association (ADA) 2014 Guidelines for target HbA1c levels per age group, namely, ≤8.5% in toddlers, ≤8% in school children, and ≤7.5% in adolescents and young adults (18).

HbA1c was measured using ion-exchange high-performance liquid chromatography technique. HbA1c values were based on measurement at regular intervals (3 months) and the average of the last 3 readings in the last year follow-up. Other variables (BP, BMI, lipid profile, and thyroid function) were recorded from the last follow-up visit.

25(OH)D measurements are done routinely for T1DM patients in our center, measured by chemiluminescent micro particle immunoassay for the quantitative determination of serum 25(OH)D in human serum and plasma. For the purpose of this study, vitamin D deficiency was defined as a serum 25(OH)D level of <37.5 nmol/L based on the Drug and Therapeutics Committee of the Lawson Wilkins Pediatric Endocrine Society (19).

### Statistical Analysis

The data were analyzed using Statistical Package for the Social Sciences version 16.5 (SPSS Inc., Chicago, IL, USA). The results were presented as percentages (%) for frequencies and means ± standard deviations for continuous variables. Independent t-test was done to compare variables that are normally distributed, and a Mann-Whitney U-test for non-Gaussian variables. Chi-square test was used to compare frequencies. Spearman correlation was done to determine associations of vitamin D status to measured variables. Linear regression using log-transformed vitamin D and HbA1c was done to determine the association between glycemic and vitamin D status. Significance was set at p-value <0.05.

## RESULTS

A total of 301 T1DM patients (161 females) were studied. Table 1 shows the clinical characteristics of males and females. The mean age for the total group was 13.9±3.8 years (13.86±3.88 for males and 14.06±3.86 for females). Mean age of T1DM at diagnosis was slightly younger in males (6.01±3.65) than females (6.33±3.45). Pubertal signs were noted in 50.7% of male and 57.8% of female subjects. Symptoms of hyperglycemia were the most common presentation (57.9% in males; 51.6% in females). Frequency of symptomatic hypoglycemic attacks was relatively higher in males (47.1%) than in females (42.9%).

Table 2 shows the anthropometric and metabolic findings and reveals that females have a slightly higher BMI than males (p=0.07). There was no significant gender difference in systolic and diastolic blood pressure, HbA1c, and lipid profile. The overall mean value for HbA1c was 9.67±1.93 (9.7±1.8 in males and 9.66±1.98 in females). Acceptable HbA1c (≤8%) values for glycemic control were found only in 26.2% (79 out of 301) of the subjects. When stratified by age, only 28.6% of toddlers, 15.6% of children, and 12.8% of adolescents had acceptable HbA1c levels. The mean level of 25(OH)D was 35.1±15.9 nmol/L. The mean 25(OH)D level was higher in males than females (36.93±14.69 versus 33.37±17.28 nmol/L; p=0.02) (Table 3).

**Figure 1:**
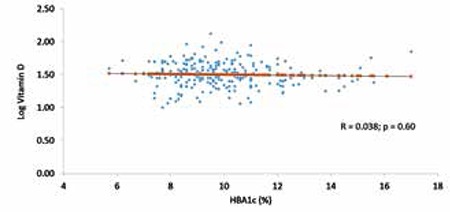
Linear correlation between log vitamin D and hemoglobin A1c

Table 3 shows the comparison of parameters according to vitamin D status and shows that subjects in the ≤37.5 nmol/L group had a significantly higher BMI and serum triglycerides than the >37.5 nmol/L group (p-values 0.019 and 0.044, respectively). In Table 4, vitamin D deficiency was noted to be 63.6% in males and 67.7% in females. In males, those who were vitamin D deficient subjects had significantly higher BMI values than those who were not deficient (p<0.01). In females, serum triglycerides were significantly higher in those who were vitamin D deficient than those who were not (p<0.05). As expected, 25(OH)D levels were higher in the sufficient than in the deficient group in both genders. There were no significant differences between the groups in the remaining parameters (Table 4). The vitamin D status of subjects was assessed for its associations to the different metabolic parameters included in the study (Table 5). In males, vitamin D status was inversely associated with frequency of hypoglycemia (p<0.01), BMI (p<0.05), diastolic BP (p<0.05), and triglyceride levels (p<0.01). In females, vitamin D status was inversely associated with age (p<0.05), age at diagnosis (p<0.01), and triglyceride levels (p<0.01). In all subjects, there was no association between circulating 25(OH)D and HbA1c (Figure 1) (r=0.04; p=0.60).

## DISCUSSION

To our knowledge, this is the first study from Saudi Arabia on the relationship between vitamin D status and metabolic control and complications in children with T1DM. The main finding of the present study is the alarmingly high prevalence of vitamin D deficiency among Saudi diabetic children and adolescents and the adverse effects of this deficiency on some cardiometabolic parameters which are gender-specific.

The high prevalence of vitamin D deficiency in our patients was an expected finding in view of the high prevalence of vitamin D deficiency in the Saudi general population, including children (20). In Saudi children, risk factors for vitamin D deficiency include gender, diet, and physical activity (21,22). Furthermore, the inverse associations of vitamin D to anthropometric and clinical parameters are in line with previous reports on non-DM, apparently healthy Saudi children (15). Nevertheless, the study supports an existing theory that expressions of vitamin D and its receptors are differentially expressed in males and females. In a recent study, differences in the modulation of different proteins at a proteomic level, associated with various pathways, including vitamin D function and activation of 1α 25(OH)D signaling, were observed to be more pronounced in males relative to females (23). This can probably explain why there were more inverse cardiometabolic associations elicited in males than the female cohort. Another explanation could be the cardioprotective effect of estrogen inherent in premenopausal women including adolescent girls (24). While vitamin D status does not seem to exert any effect in the glycemic control of our T1DM cohort, the inverse cardiometabolic associations of vitamin D in T1DM males and females warrant recommendations for vitamin D correction in this specific population. The mean age of T1DM diagnosis in Saudi Arabia, specifically in the Western region of the country, is comparatively younger than that reported for European countries, with a median age of 7.2 years in the present study (versus 6.0) (23).

There were modest gender-specific differences in Saudi patients with T1DM, with males having more symptoms than females. With regard to the clinical presentation of subjects, findings of the present study confirm the findings in previous reports from Saudi Arabia, namely, the relatively poorer metabolic control as compared to other populations, as well as the use of multiple daily insulin regimens as the most common therapy for T1DM (25).

In the present study, only 28.6% of toddlers, 15.6% of children, and 12.4% of adolescents were found to have achieved acceptable HbA1c levels indicating good glycemic control. This is an alarming situation and needs to be addressed aggressively since T1DM patients with poor metabolic control, Arab children in particular, are at highest risk for complications and cardiovascular disorders even at an early age (26,27).

Our study has some limitations. The retrospective design limits the findings to what is available in records. As such, several variables including the autoantibodies were excluded from further data analysis. Furthermore, the single-center approach limits making generalizations from the results of the study. Nevertheless, the sample size is arguably one of the largest cohorts assembled for a T1DM study in Saudi Arabia.

Metabolic control among Saudi children with T1DM is less satisfactory compared to other countries. The high prevalence of vitamin D deficiency in this population and its inverse cardiometabolic associations support the recommendation of vitamin D status correction in T1DM subjects. Further studies in a larger cohort are needed to confirm our findings.

## Figures and Tables

**Table 1:**
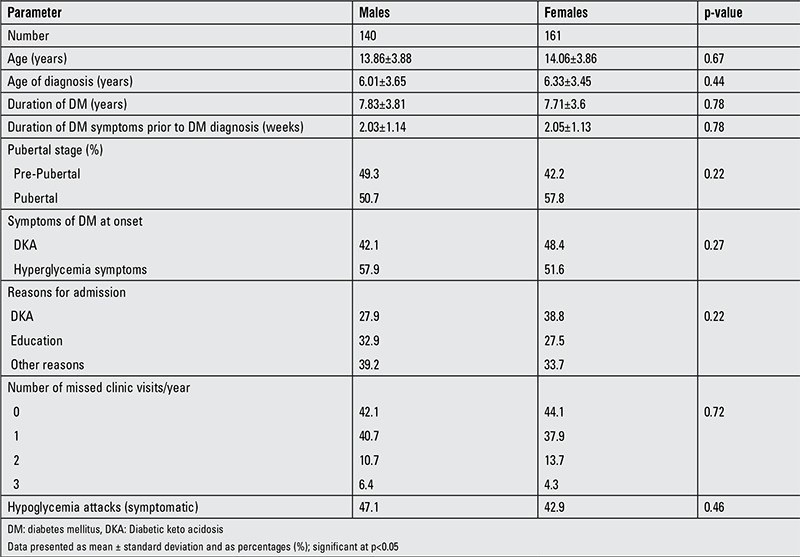
Clinical characteristics of the subjects

**Table 2:**
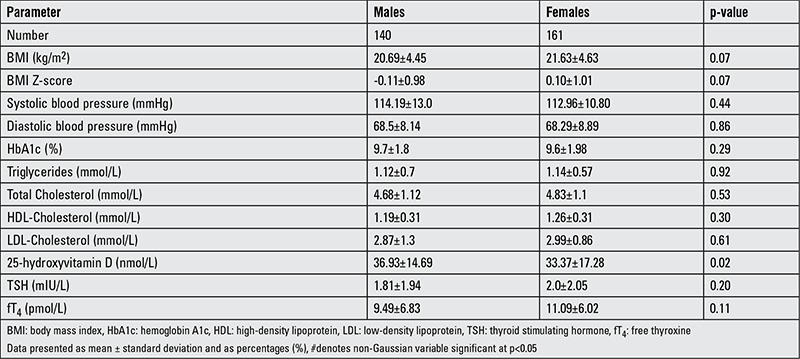
Anthropometric and metabolic characteristics of the subjects

**Table 3:**
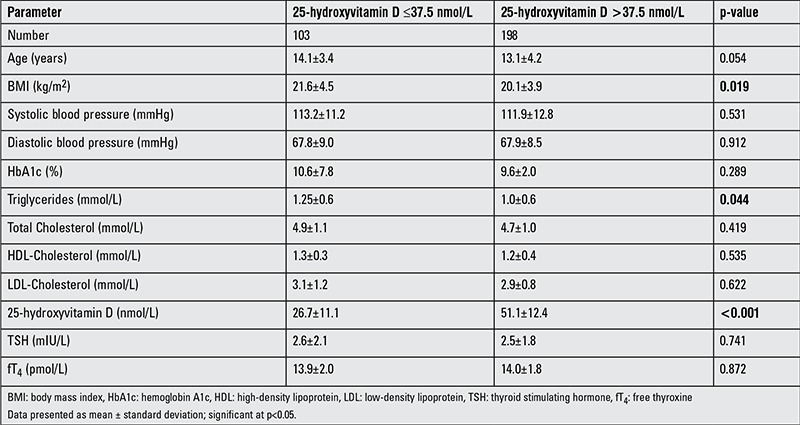
Anthropometric and metabolic characteristics by vitamin D status

**Table 4:**
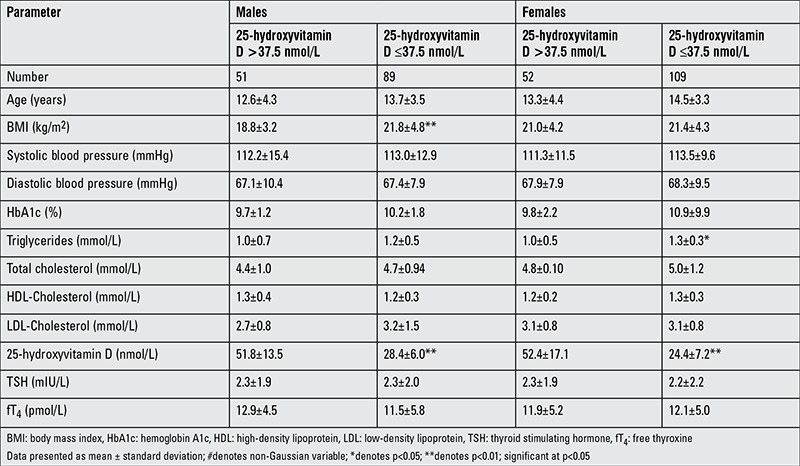
Anthropometric and metabolic characteristics by gender and vitamin D status

**Table 5:**
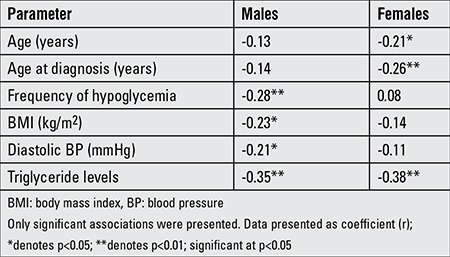
Significant correlations between 25-hydroxyvitamin D levels and clinical/metabolic parameters
